# Rhamnolipid-Enhanced ZVI-Activated Sodium Persulfate Remediation of Pyrene-Contaminated Soil

**DOI:** 10.3390/ijerph191811518

**Published:** 2022-09-13

**Authors:** Wenyang Wang, Xiyuan Wang, Hao Zhang, Qingdong Shi, Huapeng Liu

**Affiliations:** 1 College of Ecology and Environment, Xinjiang University, Urumqi 830046, China; 2 Xinjiang Key Laboratory of Oasis Ecology, Urumqi 830046, China; 3 Department of Construction and Environmental Chemical Engineering, Yanshan University Liren College, Qinhuangdao 066004, China

**Keywords:** soil washing, persulfate, ZVI, rhamnolipid, pyrene

## Abstract

In soil, polycyclic aromatic hydrocarbons (PAHs) are tightly bound to organic components, but surfactants can effectively transform them from a solid to a liquid phase. In this study, the biosurfactant rhamnolipid (RL) was selected as the eluent; shaking elution in a thermostatic oscillator improved the elution rate of pyrene, and the effects of RL concentration, temperature, and elution time on the elution effect were compared. After four repeated washings, the maximum elution rate was 75.6% at a rhamnolipid concentration of 20 g/L and a temperature of 45 °C. We found that 38 μm Zero-Valent Iron (ZVI) had a higher primary reaction rate (0.042 h^−1^), with a degradation rate of 94.5% when 3 g/L ZVI was added to 21 mM Na_2_S_2_O_8_ at 60 °C. Finally, electron paramagnetic resonance (EPR) detected DMPO-OH and DMPO-SO_4_ signals, which played a major role in the degradation of pyrene. Overall, these results show that the combination of rhamnolipid elution and persulfate oxidation system effectively remediated pyrene-contaminated soil and provides some implications for the combined remediation with biosurfactants and chemical oxidation.

## 1. Introduction

Polycyclic aromatic hydrocarbons (PAHs) [1] are organic pollutants consisting of two or more dense benzene rings produced by the combustion of fossil fuels and the disposal of industrial wastewater [2]. PAHs are common contaminants in soil and water bodies due to their carcinogenic or mutagenic properties, refractory nature, and poor volatility [3,4,5]. Therefore, it is crucial to select an appropriate remediation approach.

In recent years, advanced oxidation processes (AOPs) have been used to remediate PAHs-contaminated soils. Fenton’s reagent [6], ozone [7], permanganate [8], and persulfate (PS) are the commonly used oxidants. Moreover, PS is widely used because of its high water solubility, stability, and low cost [9,10,11]. PS can evolve under the action of transition metals as well as heat and light; additionally, the mechanism is such that sulfate radicals (SO_4_**^·^**^−^) are supplied by breaking the -O-O- bond [12,13,14]. SO_4_**^·^**^−^ has high redox potential (E^0^ = 2.4 V) [15], which makes it more capable of oxidative degradation, so many PAHs species can be degraded. PAHs in soils are characterized by low volatility and water solubility [16] and are more tightly sorbed into the soil matrix [17]; therefore, compared with PAHs in the eluent, the oxidation-only treatment of PAH-contaminated soil is time-consuming and expensive.

Surfactant elution techniques have been extensively studied to improve the migration of PAHs [18,19,20]. Surfactants lower the surface tension of water, causing PAHs to form micelles, increasing their solubility, and facilitating their dissolution from the solid to liquid phases [21]. Lominchar et al. [22] studied the effect of alkali-activated persulfate on PAHs; they discovered that adding Tween 80 to the mix enhanced the degradation rate by 30% after 25 days. In another study, Qiu et al. [23] used sodium dodecyl sulfate (SDS) to elute PAH-contaminated soil and applied Fe^2+^-activated sodium persulfate to degrade PAHs in the washing liquid. They discovered that the degradation rate increased to 60% after 10 min of reaction but remained relatively unchanged thereafter. Previous studies have shown that combining chemical surfactants elution and chemical oxidation can significantly improve remediation efficiency [24]. However, biosurfactants are considered a viable alternative to synthetic surfactants [25] due to their high biodegradability and environmental friendliness [26]. Biosurfactants are more likely to form larger micelles to increase the apparent solubility of PAHs [27]. Some of the most studied glycolipids are rhamnolipids (RLs), sucralose lipids, and locust glycolipids [28,29]. Currently, RLs are primarily used in single elution studies or in combination with bioremediation to remove organic contaminants from soils [30]. Wang et al. [31] applied various concentrations of RL to remediate DDT and PAHs in agricultural soils. Compared with the control group, the removal of both pollutants increased by 60.7 and 29.3%, respectively, but the oxidation system was not chosen for subsequent pollutant degradation in the eluate.

Pyrene is a representative of tetracyclic aromatic hydrocarbons. As there is a lack of specific studies on pyrene-contaminated soil, pyrene-contaminated soil was chosen as the research object. The novelty of this work is that rhamnolipid is first used for elution; two particle sizes of ZVI were used to activate persulfate to form SO_4_**^·^**^−^, followed by a persulfate oxidation system for eluate degradation to form a complete PAH-contaminated soil remediation system. We investigated the elution efficiency of rhamnolipids on pyrene-contaminated soil; assessed the effects of ZVI particle size, addition, Na_2_S_2_O_8_ concentration, and temperature on the degradation rate in the oxidation system; studied the morphological changes of ZVI; determined the pyrene degradation components using gas chromatography-mass spectrometry (GC-MS); and inferred the degradation pathways.

## 2. Materials and Methods

### 2.1. Materials

All the chemicals used in this study were of reagent grade. Pyrene standard (97% purity), potassium iodide (KI, 99.0%), and rhamnolipid (RL, ≥95% purity) were purchased from Shanghai McLean Biochemical Co., Ltd. (Shanghai, China). Sodium hydroxide (96% purity), sodium persulfate (Na_2_S_2_O_8_, 98% purity), and dichloromethane (99.5% purity) were purchased from Tianjin Beilian Chemical Development Co., Ltd. (Tianjin, China). Micro-ZVI (98% purity, approx. 150 μm) and micro-ZVI (98% purity, approx. 38 μm) were purchased from Aladdin Biochemical Technology Co., Ltd. (Shanghai, China). n-Hexane (98% purity), acetone (99.5% purity), and methanol (99.5% purity) were purchased from Chengdu Kolon Chemicals Co., Ltd. (Chengdu, China).

### 2.2. Supply Soil and Preparation of Contaminated Soil

Clean soil was collected at the Hongshaquan mining area in Changji, Xinjiang (88°300′–90°10′ E, 43°30′–45°00′ N) and used to prepare contaminated soil. Soil collection and preparation followed the method of Li et al. [5]: the soil was sampled at a depth of 10–20 cm, and the larger grit and rootlets were removed from the soil. The soil was left to dry naturally and passed through a 60-mesh sieve. The soil samples had a pH of 7.71, organic matter content of 2.40%, soluble salt content of 8.31 g/kg, and bulk weight of 1.1 g/cm^3^.

The contaminated soil was prepared as follows: 500 g of soil was added to 60 mg of pyrene standard dissolved in acetone, shaken, and stirred thoroughly. The spiked soil was placed in a fume hood for 40 days and stirred slowly every week. To test for the uniform distribution of pyrene, three soil samples were collected, and pyrene was extracted by sonication in dichloromethane and acetone. Finally, the pyrene content of 98 mg/kg was determined using GC-MS.

### 2.3. Solubilization and Oxidation Experiments

According to the literature [32], when combining surfactant and oxidation systems, an elution experiment should be performed first; such a sequence is conducive to experimental operation and improves repair efficiency. In the batch experiment, 2 g of pyrene-contaminated soil was placed in 50 mL conical flasks, and 20 mL of various concentrations (5, 10, 15, and 20 g/L) of rhamnolipid solution were added. Furthermore, 20 mL of ionized water was added to pyrene-contaminated soil to be used as the control. The conical flasks were sealed with Bemis sealants and placed in a THZ thermostatic shaking incubator at 160 rpm, the temperature was set at 25, 35, 45, and 55 °C, and they were removed at different shaking times (12, 24, 36, and 48 h). Finally, after standing for 20 min, 10 mL of the supernatant was recovered and placed in a centrifuge tube and centrifuged at 3500 rpm for 30 min. Two mL of the supernatant was aspirated, hexane was added for ultrasonic extraction, and the organic layer was extracted and measured using GC-MS.

The eluate was collected, the supernatant was filtered through an extractor, diluted with 30 mL deionized water, and the concentration of pyrene in the system was measured to reach 3.9 mg/L. The eluate degradation steps were as follows: 10 mL of the lysate was placed in 100 mL conical flasks, Na_2_S_2_O_8_ solution was added at various concentrations (13, 21, and 30 mM), and varying concentrations (2, 2.5, 3, and 4 g/L) of ZVI were added. The conical flasks were sealed with sealant and placed in a thermostatic shaking incubator at different temperatures (25, 40, and 60 °C) to elucidate the effect of temperature on oxidation. The rotation speed was set to 160 rpm and 3 mL of sample was taken after 10, 20, 30, 40, 50 and 60 min of reaction; to ensure that the reaction was stopped, 3 mL of methanol was added for free radical quenching. The procedure for the determination of pyrene was the same as above. Three parallel experiments were conducted for each batch to ensure the accuracy of the experiment.

### 2.4. Analytical Methods

The supernatant was passed through a 0.22 μm organic phase needle filter, and pyrene concentrations were determined using an Agilent 7890B/5977A gas chromatograph equipped with a DB-5ms 5% phenyl-methyl polysiloxane capillary column (30 m × 0.25 mm) and a full autosampler. The GC ramp-up procedure was as follows: the temperature started at 80 °C for 2 min, increased from 80 °C to 180 °C at 10 °C/min, increased from 180 °C to 280 °C at 8 °C/min, held at 280 °C for 5 min, then increased from 280 °C to 300 °C at 10 °C/min. Helium was used as the carrier gas with an injection volume of 1.0 µL. The temperature was 250 °C, and a non-split injection was used.

The concentration of RL was determined by spectrophotometry [33]; the concentration of Na_2_S_2_O_8_ in the supernatant was determined using the potassium iodide oxidation method at 352 nm [34], and the above tests were measured using an L8 UV-vis spectrophotometer (Yidian Analysis, L8, Shanghai, China). To observe the morphological changes of ZVI before and after activation, the ZVI powder was scanned using a Gemini 300 thermal field emission scanning electron microscope (Zeiss, Oxford X-MAX, Wiesbaden, Germany). The elemental species of the ZVI surface products were determined by energy spectroscopy with an X-MAX energy spectrometer (Oxford, UK), and the corrosion products were determined using Raman spectroscopy (RAMAN, LabRAM HR Evolution, Villeneuve, France).

## 3. Results

### 3.1. Optimization of RL Elution Conditions

#### 3.1.1. Effect of RL Concentration on Elution Rate

**Figure 1:**
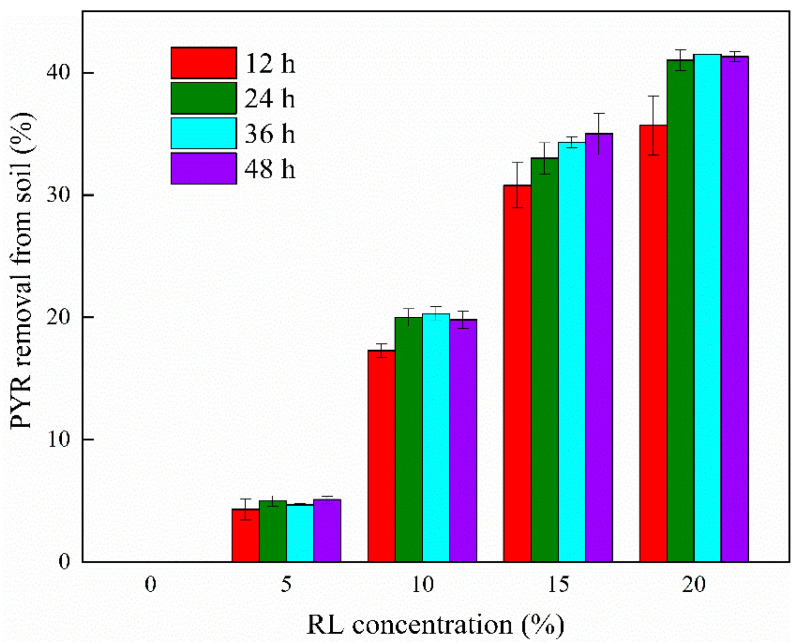
Effect of rhamnolipid concentration on elution rates. Experimental conditions: 25 °C, 160 rpm; no pH adjustment.

The effect of RL concentration on the elution rate was investigated, as shown in Figure 1. The elution rate of deionized water in the pyrene-contaminated soil was 0. This is because pyrene is insoluble in water, making it difficult for deionized water to elute pyrene from contaminated soil. The maximum elution rate increased from 5.1 to 34.3% when the concentration of RL was increased from 5 to 15 g/L because RL formed more micelles [35] when their concentration was higher than their critical micelle concentration (CMC), which reduced the (Octanol-Water Partition Coefficient, Kow) of pyrene in this system [36] and increased the solubility. However, when the concentration increased to 20 g/L, the elution rate increased by only 6.5%, and the increase in elution rate was not obvious. This was due to the fact that a high concentration of RL formed a less fluid emulsion with pyrene in an aqueous solution [37], which blocked the cracks in the soil and affected the elution of pyrene. High concentrations were difficult and costly for the oxidative degradation of the eluate; therefore, the concentration did not continue to increase in a single elution.

By observing the effect of different shaking times on the elution rate, we found that elution mainly occurred in the first 24 h at all concentrations, and the elution rate was steady in the subsequent time. Rapid solubilization may have occurred within 0–24 h [38], and then the amount of pyrene adsorbed in the RL colloid reached saturation, and pyrene dispersed from the colloid into the aqueous phase. It was difficult to increase the elution rate; therefore, 24 h was selected as the most suitable elution time.

#### 3.1.2. Effect of Temperature and Number of Drenching on Elution Rate

**Figure 2:**
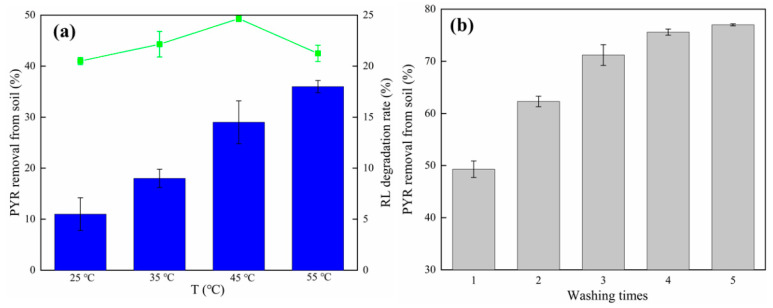
Effect of (**a**) temperature and (**b**) washing times on the elution rate of pyrene. Experimental conditions: [RL] = 20 g/L; [oscillation time] = 24 h; 25 °C, 160 rpm; no pH adjustment. RL, rhamnolipids.

As shown in Figure 2a, the elution rate of pyrene showed an upward, then a decreasing trend with increasing temperature, and reached the highest value (49.3%) at 45 °C; the elution rate then decreased by 6.8% when temperature increased to 55 °C. This is because the warming accelerated the movement of molecules in the system and increased the solubility of pyrene in the rhamnogalactan cluster [39]. During this stage, the degradation rate of rhamnolipids increased from 5.5 to 14.5%. High temperatures make it more difficult for RL molecules to form micelles and reduce the stability of the formed micelles, reducing the elution capacity of the active agent [40]. This could explain why the degradation rate of rhamnolipids continued to increase at 55 °C, but the elution rate decreased suddenly. The cumulative elution rate of pyrene gradually increased with the increasing elution rate (Figure 2b). The total elution rate was maintained at 77% after five washing cycles, while the contribution of the number of elutions to the elution effect was reduced, with only a 1.4% elution rate for the fifth cycle. This is because the principle of contaminated soil elution is the redistribution of contaminants in the liquid and solid phases [41]. When pyrene reaches partition equilibrium in the elution process, increasing the number of elutions does not effectively improve the elution rate. In addition, the remaining pyrene reacted strongly with the organic components in the soil to form PAHs [42], and it was difficult to elute the remaining pyrene by the drenching technique alone; therefore, four drenches was selected in this study.

### 3.2. ZVI Activates Na_2_S_2_O_8_ to Degrade Pyrene in Drench Solution

#### 3.2.1. Effect of ZVI Addition on Degradation Rate

**Figure 3:**
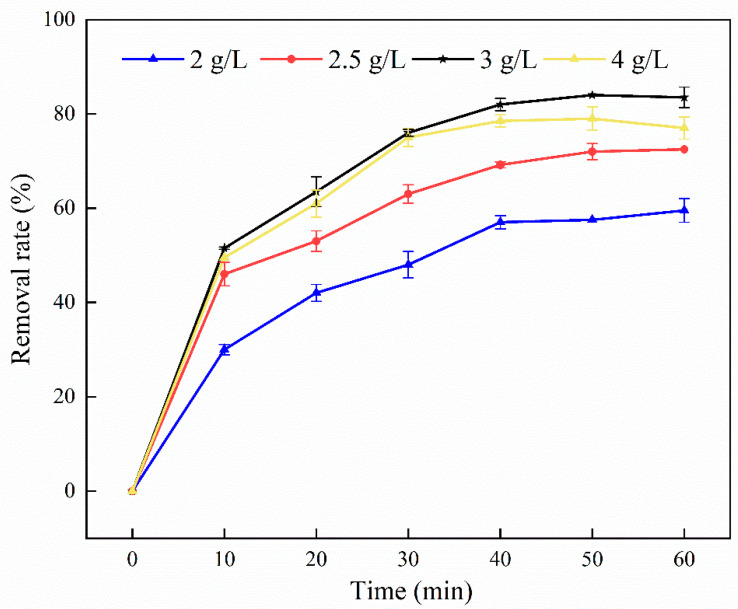
Effect of ZVI addition on degradation rate. Experimental conditions: [PS] = 21 mM; [ZVI] = 150 μm; 25 °C, 160 rpm; no pH adjustment.

First, the effect of ZVI addition on the degradation rate was explored, as shown in Figure 3. The maximum degradation rate of pyrene increased by 24.5% in 50 min by increasing the ZVI addition from 2 to 3 g/L, but the degradation rate decreased from 84 to 79% when the ZVI addition was increased to 4 g/L. For all ZVI concentrations, the degradation rate gradually increased in the first 40 min. This increase was attributed to Fe^2+^ [43] which activated Na_2_S_2_O_8_ to produce more SO_4_**^·^**^−^, increasing the degradation efficiency. After 40 min, the degradation rate remained steady, which was due to the fact that a portion of SO_4_**^·^**^−^ oxidized Fe^2+^ to Fe^3+^ (Equation (1)). Furthermore, as SO_4_**^·^**^−^ accumulated, it underwent a quenching reaction of its own free radicals [44], reducing the oxidation effect. ZVI has an optimal dose in the activation process [45,46], and when this optimal dose is exceeded, the degradation efficiency is negatively affected. Considering that 3 g/L of ZVI had the best activation effect, it was selected for the follow-up study.

Fe^2+^ + SO_4_**^·^**^−^ → Fe^3+^ + SO_4_^2−^ (1)

#### 3.2.2. Reaction Kinetics Study of Na_2_S_2_O_8_ Activation Using Two Particle Sizes of ZVI

The activation effect of persulfate differed with ZVI particle size [47] and the effect of two ZVI particle sizes (150 and 38 μm) on the degradation rate of pyrene was investigated according to the ZVI addition in Section 3.2.1. A one-stage reaction kinetic fit was performed for ZVI at the micron scale, and the degradation equation is shown in Equation (2), where C_t_ is the original concentration of pyrene in the liquid, C_0_ is the pyrene concentration at time t (min), and K_obs_ is the primary reaction rate constant (min^−1^).

(2) $$\frac{1}{C_{t}}=1+K_{\mathrm{obs}\;}C_{0}t$$

**Figure 4:**
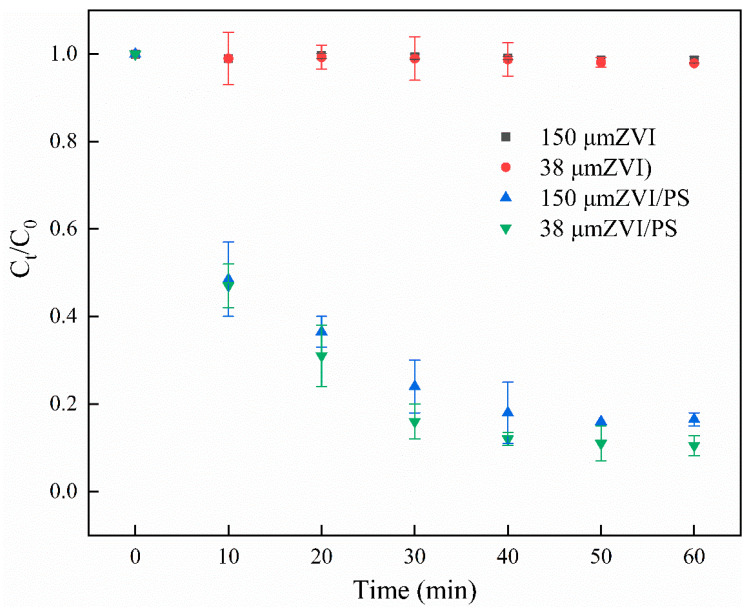
The degradation of pyrene under two sizes of ZVI. Experimental conditions: [PS] = 21 mM; [ZVI] = 3 g/L; 25 °C, 160 rpm; no pH adjustment.

The degradation rate of pyrene by ZVI is shown in Figure 4. The highest degradation rate of pyrene by 38 μm ZVI was only 2.1%, and the effect of ZVI addition on the degradation rate increased and then decreased. After 60 min of reaction with ZVI addition of 3 g/L, Na_2_S_2_O_8_ concentration of 21 mM, and temperature of 25 °C, the degradation rate of 38 μm ZVI-activated eluate was 89.5%, and the degradation rate of 150 μm ZVI was 83% under the same reaction conditions. The kinetics of the primary degradation of pyrene were fitted to Figure S1, and reaction rate constants of 0.030 and 0.042 h^−1^ were found for pyrene after the activation of both ZVIs, indicating that 38 μm ZVI was more effective in activating Na_2_S_2_O_8_. This is consistent with the findings of Li et al. [48]; they found that the smaller particle size of ZVI powder had better removal efficiency for AO7, which was attributed to the higher specific surface area of ZVI with smaller particle size. The smaller particle size of ZVI improved the reaction rate of the reactive group SO_4_**^·^**^−^ [49]; therefore, the 38 μm ZVI was chosen for this experiment.

#### 3.2.3. Effect of Na_2_S_2_O_8_ Concentration and Temperature on the Degradation Rate

**Figure 5:**
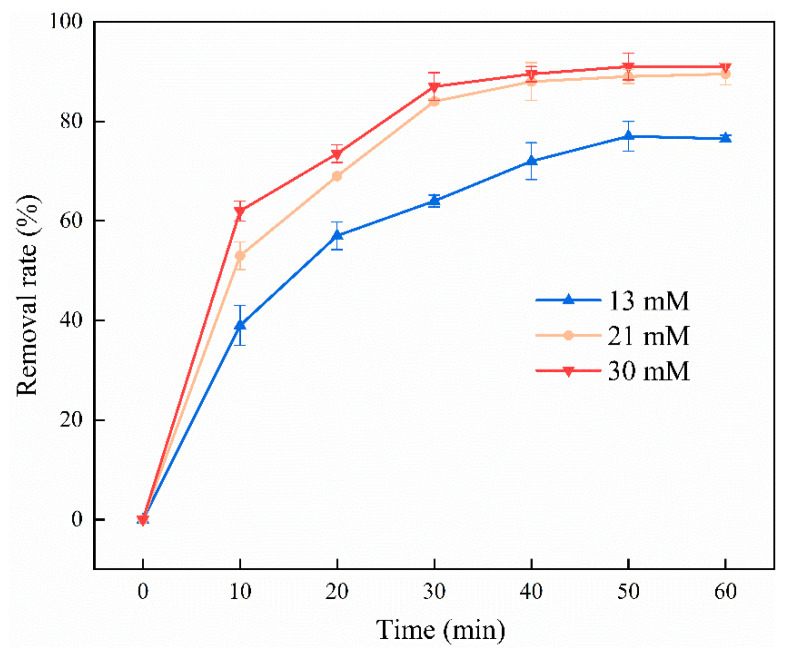
Effect of persulfate concentration on the degradation of pyrene. Experimental conditions: [ZVI] = 3 g/L; 25 °C; 160 rpm; no pH adjustment.

Because persulfate is the source of SO_4_**^·^**^−^ production [50], persulfate concentration is a critical factor in the persulfate oxidation system. When the concentration was increased from 13 and 21 to 30 mM, the degradation rate of pyrene increased from 72 and 88, respectively, to 90%, after 40 min of reaction (Figure 5). The slow increase in degradation rate was because Fe^2+^ was the decisive factor for the SO_4_**^·^**^−^ production. The production of Fe^2+^ was close to saturation, and it was difficult to produce more SO_4_**^·^**^−^ during activation. For all concentrations, the degradation rate was steady after 40 min. This may be due to the fact that RL decomposed to **·**OH, and the SO_4_**^·^**^−^ generated by activation was quenched by S_2_O_8_^2−^ and **·**OH [51], leading to a decrease in the degradation rate.

The effect on the oxidation system was determined by adjusting the temperature (25, 40, and 60 °C). Figure S2 shows that the maximum degradation rate of pyrene increased from 89 to 94.5% when the temperature was increased from 25 to 60 °C; the increase in temperature effectively reduced the time required for the reaction to reach equilibrium. There were two main reasons for the increase in degradation rate with temperature: (1) The reaction to generate SO_4_**^·^**^−^ was a heat-absorbing reaction, and increasing the external temperature was conducive to increasing the confusion at the interface between Na_2_S_2_O_8_ and pyrene [52], which sped up the reaction process. (2) In addition to the metal, warming also produces activation, and this reaction forms a two-way activation [53], accelerating the production of SO_4_**^·^**^−^. Continuing to increase the temperature would be more costly, so 60 °C was chosen as the appropriate temperature.

#### 3.2.4. Analysis of Surface Morphology Changes and Reaction Products of ZVI

**Figure 6:**
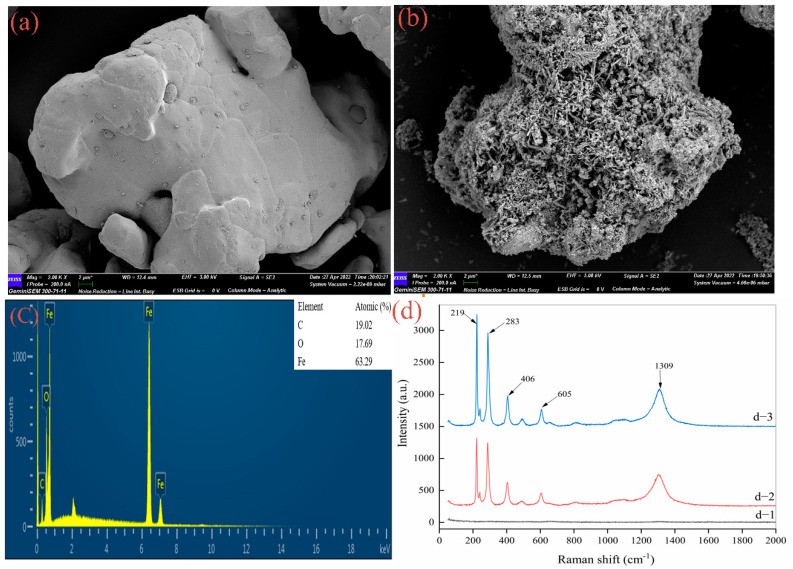
SEM and EDS images before and after the 38 μmZVI reaction. (**a**) SEM image of unreacted ZVI, (**b**) SEM image of ZVI after reaction, (**c**) EDS image of after reaction, and (**d**) Raman spectra before and after the reaction. [d − 1] 38 μmZVI; [d − 2] 38 μmZVI/PS; [d − 3] 38 μmZVI/PS/elution solution.

Scanning electron microscopy (SEM) and energy dispersive spectroscopy (EDS) can clearly observe the morphological changes of ZVI before and after the reaction [54,55], identify the elemental species of the products, and effectively characterize iron oxides on the ZVI surface under different reaction conditions [56]; therefore, the above tools were chosen in this section. As shown in Figure 6a, the surface of ZVI that was not involved in the reaction was smooth and flat, with only a few particles attached. After participating in the reaction, ZVI corroded, its surface generated more holes and cracks, and it was attached to filamentous substances (Figure 6b). Figure 6c shows the EDS peak spectra with the characteristic peak of O element; its content accounted for 17.69%, indicating that ZVI produced oxidized substances after the reaction. Raman spectra analysis of ZVI after the reaction showed strong spectral bands at 219, 283, and 1309 cm^−1^. According to the literature [57], the strong narrow bands at 219 and 283 cm^−1^ corresponded to the formation of α-Fe_2_O_3_, and the spectral band at 1309 cm^−1^ belonged to the second-order scattering of α-Fe_2_O_3_. Li et al. [58] showed that the characteristic peak at 406 cm^−1^ belonged to α-FeOOH and considered the low band at 605 cm^−1^ to be a mixture of α-Fe_2_O_3_ and α-FeOOH. Therefore, the main corrosion products of the 38 μm-ZVI were α-Fe_2_O_3_ and a small amount of α-FeOOH (Figure 6d).

#### 3.2.5. Possible Intermediates and Approaches of Pyrene Degradation

**Figure 7:**
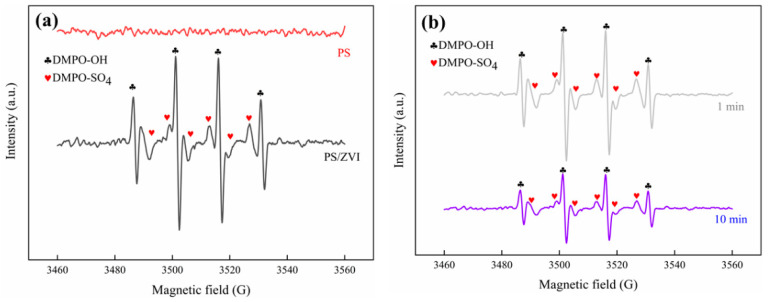
EPR spectra: (**a**) PS single and adopting ZVI/PS system; and (**b**) at various reaction times under ZVI/PS system. Experimental conditions: [PS] = 10 mM; [ZVI] = 1 mg/mL; [DMPO] = 50 mM; no pH adjustment.

Pyrene decomposes into volatile and semi-volatile oxygenated organic compounds by oxidation; the concentration of these organics is often very high and composition is complex [59]. Thus, inferring the degradation pathways and main products of pyrene is crucial for the treatment and application of water bodies. The PS/ZVI system acted mainly through SO_4_**^·^**^−^ and **·**OH radicals in the degradation of pyrene [60]. This study performed EPR analysis with DMPO as a complementary agent to verify the presence and content changes of SO_4_**^·^**^−^ and **·**OH radicals. In the absence of ZVI, no signals related to free radicals were obtained (Figure 7a), indicating that the production of SO_4_**^·^**^−^ and **·**OH radicals by a single PS was difficult. After 1 min of ZVI addition, DMPO-**·**OH (1:2:2:1) and DMPO-SO_4_ (1:1:1:1:1:1) showed the presence of SO_4_**^·^**^−^ and **·**OH radicals, and after 10 min, there was a significant decrease in the SO_4_**^·^**^−^ and **·**OH contents (Figure 7b) because of the consumption of SO_4_**^·^**^−^ and **·**OH by the oxidation [61].

**Figure 8:**
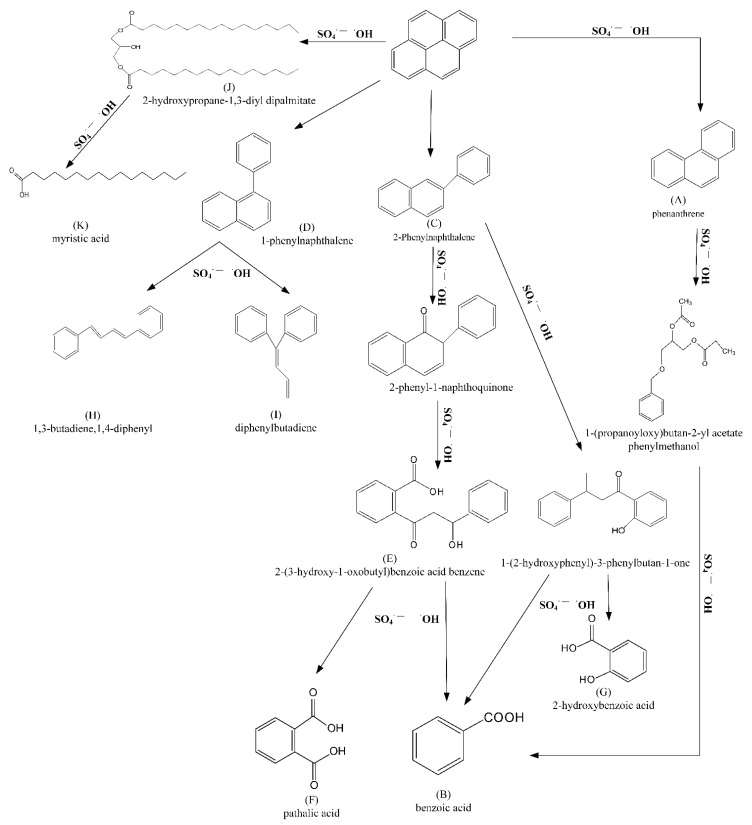
Degradation pathways of pyrene in persulfate oxidation systems.

GC-MS identified the main decomposition products of pyrene in the persulfate oxidation system, as summarized in Table S1: phenanthrene, 2-phenylnaphthalene, 2-hydroxypropane-1,3-diyl dipalmitate, 1,3-butadiene,1,4-diphenyl, and diphenylbutadiene were the main intermediates. On the basis of these intermediates and chemical mechanisms, the main degradation pathways of pyrene are shown in Figure 8. Four main pathways were involved in the initial attack of pyrene. First, pyrene was converted to phenanthrene (A) under attack by SO_4_**^·^**^−^ and **·**OH [62] and finally converted to benzoic acid (B). In the second pathway, pyrene first underwent rearrangement to 2-phenylnaphthalene (C) and 1-phenylnaphthalene (D), and C was oxidized by SO_4_**^·^**^−^ and **·**OH to the intermediate product 2-(3-hydroxy-1-oxobutyl) benzoic acid benzene (E); intermediate products were converted to short-chain carboxylic acids [63], benzoic acid (B), and pathalic acid (F) in the presence of SO_4_**^·^**^−^ and **·**OH; in addition, under attack by free radicals, pyrene also directly produced intermediate 1-(2-hydroxyphenyl)-3-phenylbutan-1-one, which was subsequently oxidized to benzoic acid (B) and 2-hydroxybenzoic acid (E) [64]. The third oxidation pathway occurred when the oxidation capacity of SO_4_**^·^**^−^ and **·**OH was weakened, and in the presence of free radicals, 1-phenylnaphthalene (D) was converted into 1,3-butadiene,1,4-diphenyl (H), and diphenylbutadiene (I), at the point where the oxidation capacity was already poor. Cleavage and decarboxylation of the aromatic ring occurred when the benzene ring was attacked by free radicals by converting the macromolecule substance 2-hydroxypropane-1,3-diyl dipalmitate (J) and then oxidizing it to myristic acid (K), which was the last pathway.

## 4. Conclusions

In this study, the pyrene-contaminated soil was first eluted with rhamnolipids, and then ZVI-activated Na_2_S_2_O_8_ was selected for the degradation of pyrene in the eluate. Morphological changes before and after the ZVI reaction were observed, and the degradation pathway was inferred based on GC-MS. The elution rate was found to be positively influenced by the concentration of rhamnolipids and the number of elutions, but high temperature reduced the elution effect, and the highest elution rate was 75.6%. When Na_2_S_2_O_8_ was activated by ZVI with two particle sizes, the activation effect of the 38 μm ZVI was better under the reaction conditions of 3 g/L ZVI addition, 30 mM Na_2_S_2_O_8_ concentration, and 60 °C, and the degradation rate of pyrene reached 94.5% in 40 min. SEM scanning and Raman spectra showed that α-Fe_2_O_3_ was the main corrosion product of the 38 μmZVI. In addition, 11 major decomposition substances were detected by GC-MS, and the main pathways for pyrene degradation were divided into four parts. The effect of nanoscale ZVI on activation was not compared in this study, which was a limitation. The above results indicated that rhamnolipid elution combined with the SO_4_**^·^**^−^ oxidation system has good application prospects for PAH-contaminated soil.

## 5. Discussion

Since the goal of this investigation was to explore the possibilities of cooperative remediation, artificially contaminated soil was chosen as the study object. However, due to variations in physicochemical qualities, that was distinct from actual contaminated soil. With more types of PAHs in real soils, the elution effect of surfactants may be reduced.

In addition, in the oxidation experiments of the eluate, more sizes of ZVI were not selected for the activation of Na_2_S_2_O_8_, nor did we consider the economic cost of applying this experiment to real contaminated soil, which was also a limitation of this study.

In the future, researchers should apply more experimental results to actual contaminated soil, and hopefully, this study can provide suggestions for remediation of organic contaminated soil.

## Data Availability

Not applicable.

## Supplementary Materials

The following supporting information can be downloaded at: https://www.mdpi.com/article/10.3390/ijerph191811518/s1. Figure S1: degradation kinetics. Reaction conditions: [PS] = 21 mM; [ZVI] = 3 g/L; 25 °C, 160 rpm; no pH adjustment. Figure S2: Effect of temperature on the degradation of pyrene. Experimental conditions: [PS] = 21 mM; [ZVI] = 3 g/L; 160 rpm; no pH adjustment. Table S1: Determination of possible intermediates of pyrene degradation by GC-MS.
