# Supplementary material for: Rhamnolipid-Enhanced ZVI-Activated Sodium Persulfate Remediation of Pyrene-Contaminated Soil
Source: Int J Environ Res Public Health. 2022 Sep 13;19(18):11518. doi: 10.3390/ijerph191811518 (PMC9517034; doi:10.3390/ijerph191811518)
Supplement: Supplementary file 1 [file ijerph-19-11518-s001.zip › ijerph-1884281-supplementary.pdf]

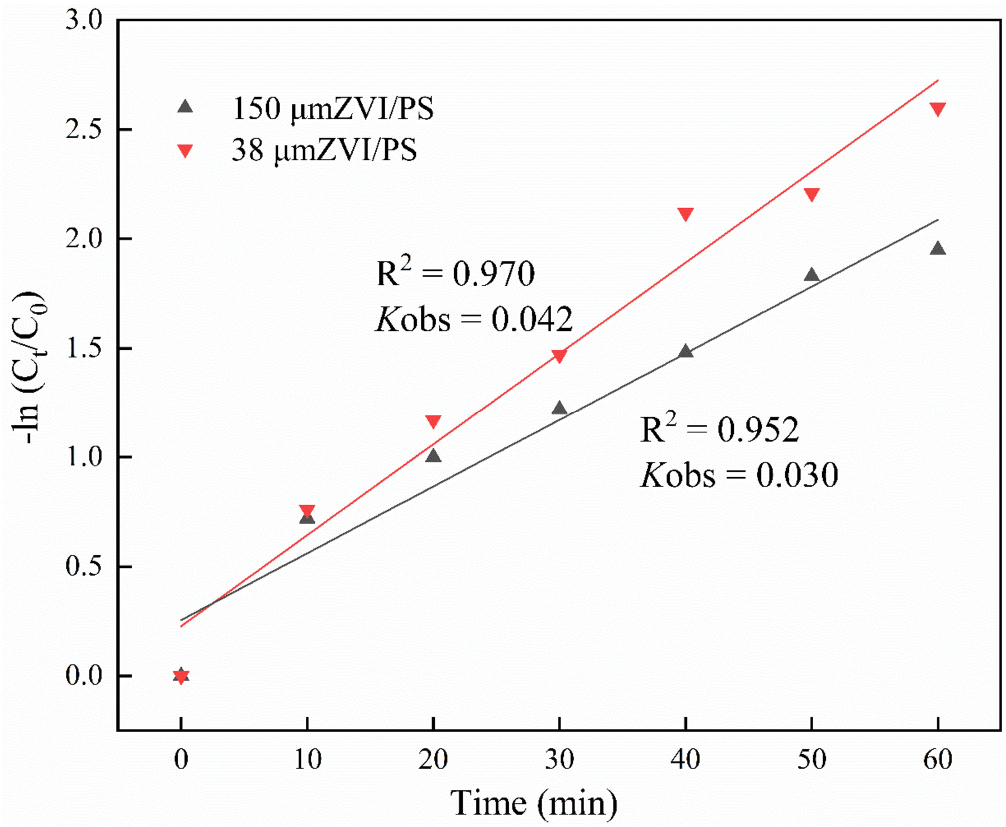

Figure S1. degradation kinetics. Experimental conditions: [PS] = 21 mM; [ZVI] = 3 g/L ;25 ℃, 160 rpm; no pH adjustment.

Micron-sized particles of ZVI (150 and 38 μm) follow the primary reaction kinetic equation, so our study applies to the primary reaction dynamics equation. The equation is given as follows:

$$-\ln\frac{C_t}{C_0} = K_{obs}\,t$$

The vertical coordinate is $-\ln\frac{C_t}{C_0}$, the horizontal coordinate is t, and the slope is K_{obs} .

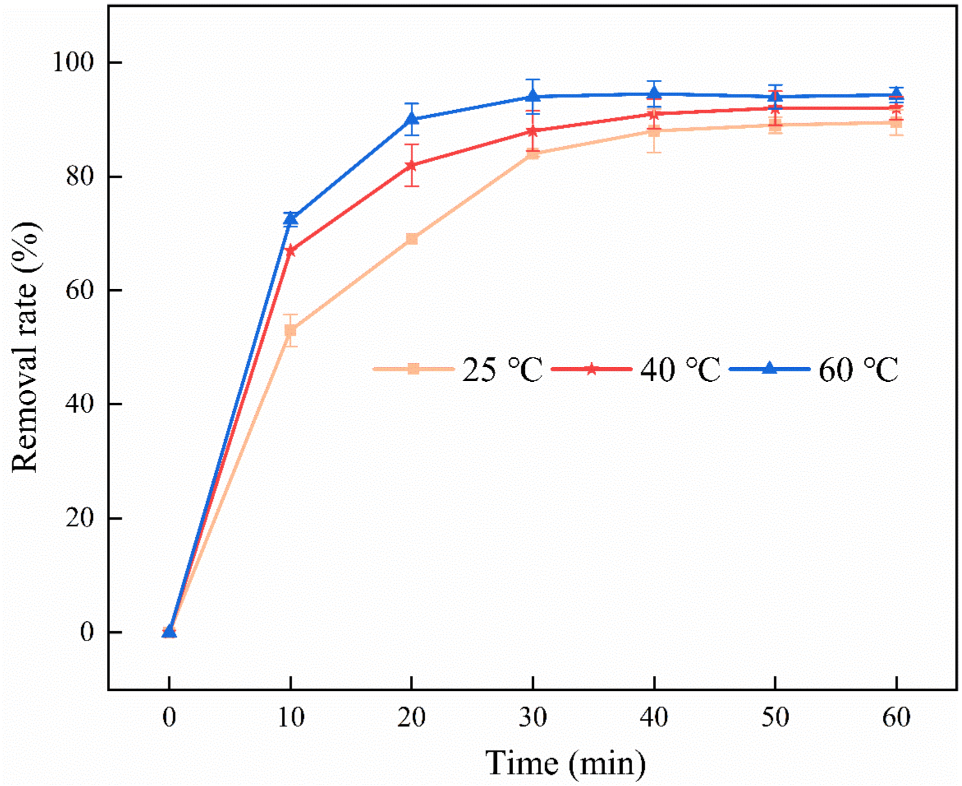

Figure S2. Effect of temperature on the degradation of pyrene. Experimental conditions: [PS] = 21 mM; [ZVI] = 3 g/L; 160 rpm; no pH adjustment.

Table S1. Determination of possible intermediates of pyrene degradation by GC-MS.

Products	Chemical name	Structure	R. Time (min)
A	phenanthrene	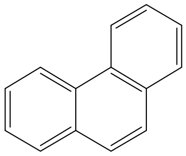	18.172
B	benzoic acid	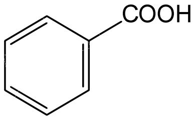	6.694
C	2-phenylnaphthalene	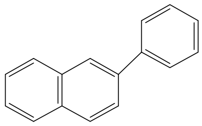	13.280

D	1-phenylnaphthalene	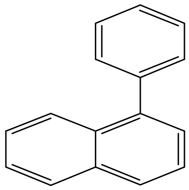	13.280
E	2-(3-hydroxy-1-oxobutyl) benzoic acid benzene	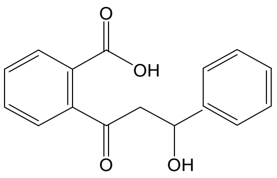	11.465
F	pathalic acid	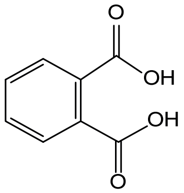	10.152
G	2-hydroxybenzoic acid	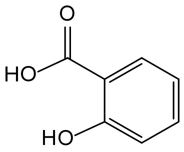	7.291
H	1,3-butadiene,1,4-diphenyl	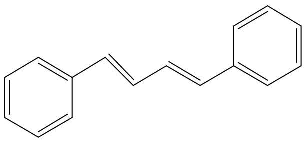	12.670
I	diphenylbutadiene	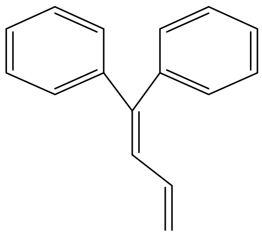	12.250
J	2-hydroxypropane-1,3-diyl dipalmitate	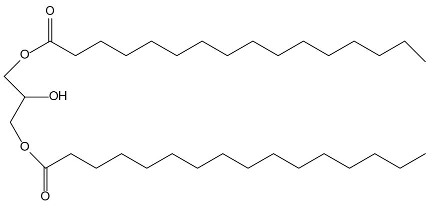	24.125
K	myristic acid	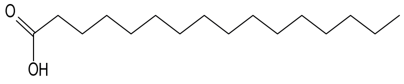	13.615
